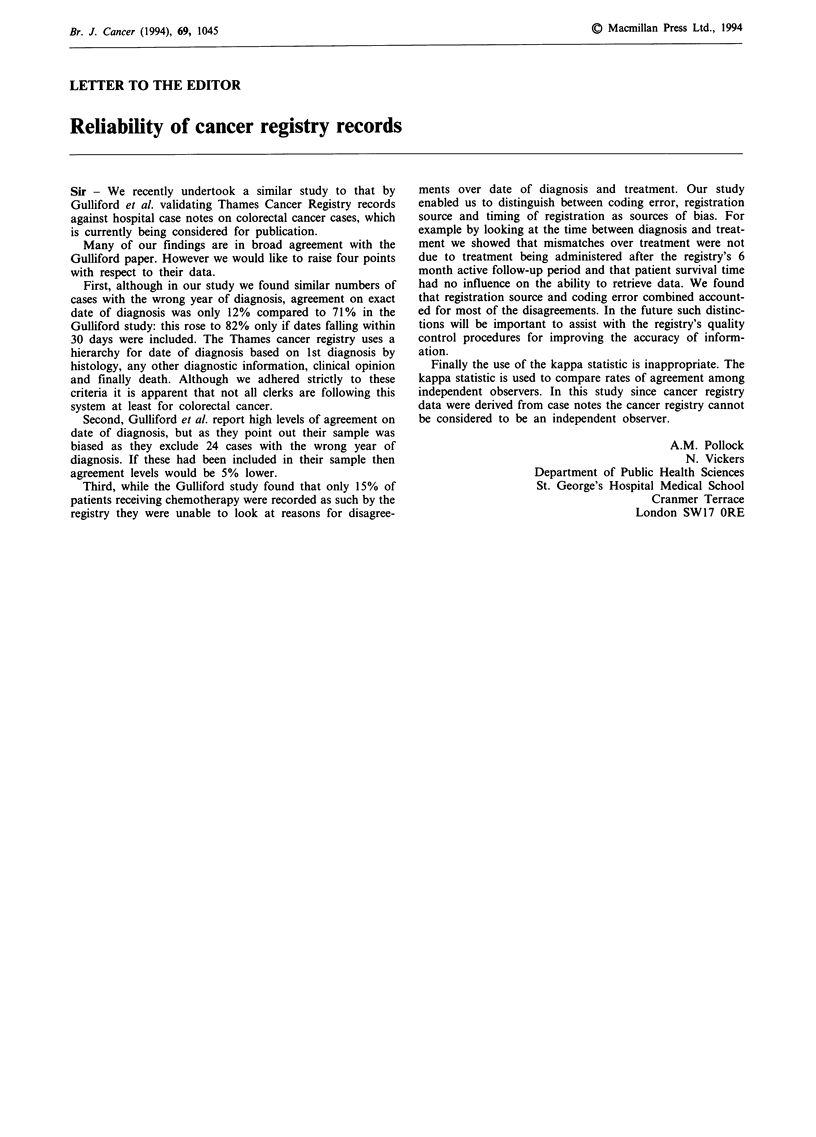# Reliability of cancer registry records.

**DOI:** 10.1038/bjc.1993.479

**Published:** 1993-11

**Authors:** A. M. Pollock, N. Vickers


*Br. J. Cancer* (1994), **69**, 1045 © Macmillan Press Ltd., 1994

LETTER TO THE EDITOR

### Reliability of cancer registry records

**Sir** – We recently undertook a similar study to that by Gulliford *et al.* validating Thames Cancer Registry records against hospital case notes on colorectal cancer cases, which is currently being considered for publication.

Many of our findings are in broad agreement with the Gulliford paper. However we would like to raise four points with respect to their data.

First, although in our study we found similar numbers of cases with the wrong year of diagnosis, agreement on exact date of diagnosis was only 12% compared to 71% in the Gulliford study: this rose to 82% only if dates falling within 30 days were included. The Thames cancer registry uses a hierarchy for date of diagnosis based on 1st diagnosis by histology, any other diagnostic information, clinical opinion and finally death. Although we adhered strictly to these criteria it is apparent that not all clerks are following this system at least for colorectal cancer.

Second, Gulliford *et al.* report high levels of agreement on date of diagnosis, but as they point out their sample was biased as they exclude 24 cases with the wrong year of diagnosis. If these had been included in their sample then agreement levels would be 5% lower.

Third, while the Gulliford study found that only 15% of patients receiving chemotherapy were recorded as such by the registry they were unable to look at reasons for disagreements over date of diagnosis and treatment. Our study enabled us to distinguish between coding error, registration source and timing of registration as sources of bias. For example by looking at the time between diagnosis and treatment we showed that mismatches over treatment were not due to treatment being administered after the registry's 6 month active follow-up period and that patient survival time had no influence on the ability to retrieve data. We found that registration source and coding error combined accounted for most of the disagreements. In the future such distinctions will be important to assist with the registry's quality control procedures for improving the accuracy of information.

Finally the use of the kappa statistic is inappropriate. The kappa statistic is used to compare rates of agreement among independent observers. In this study since cancer registry data were derived from case notes the cancer registry cannot be considered to be an independent observer.

A.M. Pollock
N. Vickers
Department of Public Health Sciences
St. George's Hospital Medical School
Cranmer Terrace
London SW17 0RE